# Changes in the oxidative status and damage by non-essential elements in the digestive gland of the gastropod *Pomacea canaliculata*

**DOI:** 10.3389/fphys.2023.1123977

**Published:** 2023-03-22

**Authors:** Alejandra D. Campoy-Diaz, Gabriela Malanga, Maximiliano Giraud-Billoud, Israel A. Vega

**Affiliations:** ^1^ IHEM—CONICET, Universidad Nacional de Cuyo, Mendoza, Argentina; ^2^ Facultad de Ciencias Médicas, Instituto de Fisiología, Universidad Nacional de Cuyo, Mendoza, Argentina; ^3^ Departamento de Ciencias Básicas, Escuela de Ciencias de la Salud-Medicina, Universidad Nacional de Villa Mercedes, San Luis, Argentina; ^4^ Facultad de Farmacia y Bioquímica, Fisicoquímica, Universidad de Buenos Aires, Buenos Aires, Argentina; ^5^ Instituto de Bioquímica y Medicina Molecular (IBIMOL), CONICET—Universidad de Buenos Aires, Buenos Aires, Argentina; ^6^ Departamento de Biología, Facultad de Ciencias Exactas y Naturales, Universidad Nacional de Cuyo, Mendoza, Argentina

**Keywords:** oxidative stress, antioxidant defense, water pollution, arsenic, mercury, uranium, oxidative damage, apple snail

## Abstract

The freshwater gastropod *Pomacea canaliculata* fulfills the ideal conditions of a bioindicator species since its digestive gland bioconcentrates elements toxic for human and ecosystems health. The aim of this work was to study the balance between production of free radicals and antioxidant defenses, and the generation of oxidative damage in the digestive gland of this mollusk after exposure (96 h) to three elements with differential affinities for functional biological groups: mercury (5.5 μg/L of Hg^+2^ as HgCl_2_), arsenic [500 μg/L of (AsO_4_)^−3^ as Na_3_AsO_4_7H_2_O], or uranium [700 μg/L of (UO_2_)^+2^ as UO_2_(CH_2_COOH)_2_]. Bioconcentration factors of Hg, As, and U were 25, 23, and 53, respectively. Snails exhibited a sustained increase of reactive species (RS), and protein and lipid damage. Lipid radicals increased between 72 and 96 h, respectively, in snails exposed to U and Hg while this parameter changed early (24 h) in As- exposed snails. Snails showed protein damage, reaching maximum values at different endpoints. This redox disbalance was partially compensated by non-enzymatic antioxidant defenses α-tocopherol (α-T), β-carotene (β-C), uric acid, metallothionein (MTs). Snails consumed α-T and β-C in an element-dependent manner. The digestive gland consumed rapidly uric acid and this molecule was not recovered at 96 h. Digestive gland showed a significant increase in MTs after elemental exposure at different endpoints. The enzymatic antioxidant defenses, represented by the catalase and glutathione-S-transferase activities, seems to be not necessary for the early stages of the oxidative process by metals. This work is the first attempt to elucidate cellular mechanisms involved in the tolerance of this gastropod to non-essential elements. The bioconcentration factors and changes in the oxidative status and damage confirm that this species can be used as a bioindicator species of metal pollution in freshwater bodies.

## 1 Introduction

The generation of prooxidant molecules is a consequence of normal cellular functioning (Sies et al., 2017). Eukaryotic cells produce enzymatic and non-enzymatic antioxidant defenses to avoid the oxidative damage that prooxidants could cause in macromolecule (Sies, 1993; Stahl and Sies, 2003). Several external factors can promote an enhanced prooxidant generation (Sies et al., 2017). Previous investigations have shown that metals increase the generation of reactive oxygen and nitrogen species (RS), thus affecting cellular oxidative homeostasis and leading to oxidative imbalance, commonly known as oxidative stress (for a review see Valko et al., 2005; 2007). The oxidative imbalance can damage cells or tissues by lipid peroxidation, protein carboxylation, and even modifications in the DNA (Newman and Unger, 2003).

The apple snail *Pomacea canaliculata* has emerged as a possible bioindicator of elemental pollution (Adewunmi et al., 1996; Ezemonye et al., 2006; Deng et al., 2008; Vega et al., 2012; Campoy-Diaz et al., 2018; Campoy-Diaz et al., 2020) due to its worldwide distribution in humid tropical and subtropical freshwater ecosystems (Hayes et al., 2015), can be cultured and kept under laboratory-controlled conditions, and their tissues can accumulate a diverse array of ecologically relevant elements (e.g. As, Cd, Cu, Hg, Pb, U, Zn) (Callil and Junk, 2001; Peña and Pocsidio, 2008; Vega et al., 2012; Campoy-Diaz et al., 2018; Huang et al., 2018; Campoy-Diaz et al., 2020; Juarez et al., 2022). Furthermore, the digestive gland and symbionts from *P. canaliculata* participate in metal accumulation. Additionally, these symbionts are involved in metal depuration (Castro-Vazquez et al., 2002; Vega et al., 2005; Vega et al., 2006; Vega et al., 2012; Campoy-Diaz et al., 2018; Campoy-Diaz et al., 2020) when are expelled in the feces (Koch et al., 2006). However, our knowledge about the physiological response of the digestive gland to different prooxidant elements remains incomplete.

The high persistence, wide environmental occurrence, and proven toxicity of metals make them an urgent issue requiring priority study. Nieboer and Richardson (1980) have emphasized the biochemical basis for metal-ion toxicity, together with the ecotoxicological and ecophysiological significance for organisms and ecosystems exposed to these elements. They classify metals by their differential affinities for functional biological groups: A class elements seek oxygen (O), B class elements seek nitrogen (N) and sulfur (S), and intermediate class elements seek either O, N, or S. In this study, we have selected three elements included in the first 100 substances of the priority list of Agency for Toxic Substances and Disease Registry: U from the A-class, Hg from the B-class, and As from the intermediate-class. By including these elements with different biochemical affinities for biomolecules, we evaluated the balance between free radicals’ production and antioxidant response, as well as oxidative damage in the digestive gland of *P. canaliculata* after elemental exposure.

## 2 Materials and methods

### 2.1 Animals and rearing conditions

Adult individuals of 4 months old (shell length = 34.8–40.3 mm) from a cultured strain of *P. canaliculata* were used (Giraud-Billoud et al., 2011). Snails were maintained in aquaria containing 6 L of tap water at 23°C–26°C in presence of artificial lighting (14 h per day). These culturing conditions were approved by the Institutional Animal Care and Use Committee of the Universidad Nacional de Cuyo, Facultad de Ciencias Médicas (Protocol N 55/2015). Adult snails were only fed with lettuce in all experimental sets (Sections 2.2 and 2.3).

### 2.2 First experimental set: Elemental bioconcentration by neutron activation analysis

An experiment was initially run to know whether Hg, As, and U, are accumulated in the digestive gland after acute exposure (96 h), compared to non-exposed snails. Sixteen animals were divided into four experimental groups of four snails (one separate aquarium per group). Each snail group was exposed to either (a) culturing water (control), (b) 5.5 μg/L of Hg as HgCl_2_ (Sigma-Aldrich, M1136; purity 99%), (c) 500 μg/L of As as Na_3_AsO_4_7H_2_O (Sigma-Aldrich, S9663; purity ≥98%), or (d) 700 μg/L of U as UO_2_(CH_2_COOH)_2_ (Ted Pella Inc.19481). These concentrations correspond to maximum levels detected in aquatic environments registered for the World Health Organization: Hg in wells from Izu Oshima volcanic Island, Japan (WHO, 2005), As in anthropogenic sources and in geothermal waters (WHO, 2011), and U in private supplies in Canada (WHO, 2012). After exposure, we carefully cracked the shell, dissected, sampled the digestive gland (∼100 mg), and then these individual samples were lyophilized and stored at −80°C (Vega et al., 2012). The three elements were analyzed by neutron activation using a RA-3 reactor. Elemental concentration was calculated according to Kolthoff and Elvin (1986), using the absolute parametric method current tables. In order to check the accuracy of the method, four certified reference materials (NRCC-DORM2, NRCC-TORT, IAEA-MA-A-2, and IAEA-140/TM) were analyzed together with the samples for the determination of Hg and As (see Supplementary Material from Campoy-Diaz et al., 2018). For uranium, NIST-2704 and IAEA-SL1 were analyzed (see Supplementary Material from Campoy-Diaz et al., 2018). Elemental concentrations were expressed as milligram per kilogram of dry weight. The raw data is reported in Supplementary Data S1.

### 2.3 Second experimental set: Changes in the oxidative state and oxidative damage

A second experiment was conducted to find out if Hg, As, and U produce changes in the oxidative state and damage in the digestive gland during the first 96 h after elemental exposure, compared to unexposed snails. Eighty-five animals were divided into seventeen aquariums (N = 5 individuals each). The whole experimental design included: (a) five aquariums exposed to culturing water for 0, 24, 48, 72, and 96 h, (b) four aquariums exposed to 5.5 μg/L of Hg for 24, 48, 72, and 96 h, (c) four aquariums exposed to 500 μg/L of As for 24, 48, 72, and 96 h, and (d) four aquariums exposed to 700 μg/L of U for 24, 48, 72, and 96 h. No snail deaths were recorded. Snails were euthanized at each endpoint (0, 24, 48, 72, and 96 h), and samples of digestive gland were collected and processed individually for further studies of oxidative stress (Section 2.3.1), non-enzymatic (Sections 2.3.2), and enzymatic (Section 2.3.3) antioxidant defenses, and oxidative damage (Sections 2.3.4 and 2.3.5). The raw data is reported in Supplementary Data S2.

#### 2.3.1 Reactive oxygen and nitrogen species production

The production of RS was measured through the oxidation of 2′,7′-dichlorofluorescein diacetate (DCFH-DA) (Wang and Joseph, 1999; Malanga et al., 2014). Tissue samples (∼40 mg) were homogenized (1:5 w/v) in 100 mM Tris-HCl buffer (pH 7.75) containing 5 mM MgCl_2_ and 2 mM EDTA. The homogenates were centrifuged at 4°C at ×100,00 *g* for 20 min and the obtained supernatants were incubated with 40 mM DCFH-DA in a buffered solution (30 mM HEPES, 200 mM KCl, 1 mM MgCl_2_, pH 7.2) for 15 min at 37°C. First, supernatant esterase hydrolyzed DCFH-DA forming DA and DCFH. Then, the latter was oxidized by RS forming a fluorescent compound DCF, which was detected at *λ* excitation = 485 nm and *λ* emission = 538 nm wavelengths. Results were expressed as arbitrary units of fluorescence (AUF) per milligram of wet weight, generated in a min.

#### 2.3.2 Non-enzymatic antioxidant defenses: α-tocopherol (α-T), β-carotene (β-C), metallothioneins (MTs), and uric acid

The α-T and β-C concentrations were identified and then quantified by reverse-phase HPLC with electrochemical detection using a Bioanalytical Systems LC-4C amperometric detector and a glassy carbon-working electrode at an oxidation potential of 0.6 V (Desai, 1984). Digestive gland samples (∼35 mg) were homogenized by sonication in 100 µL of distilled water and 15 µL of butylated hydroxytoluene (BHT) 4% (W/V). The organic phase was extracted with 200 µL of methanol and 900 µL of hexane. After centrifugation at ×6,000 *g* for 5 min, the hexane phase was removed and evaporated to dryness under N_2_. The remainders were dissolved in methanol/ethanol (1:1) and injected for isocratic HPLC analysis (Desai, 1984), Waters 510 bomb, Supelcosil LC-8 column (15 cm × 4.6 mm, 3 µm particle size). The flow was 1 ml/min using a solution of methanol:water (99:1) containing 20 mM lithium perchlorate as mobile phase. The characteristic retention times ranged respectively from 3.057 to 3.167 min and 4.930–5.153 min for α-T and β-C. Stock solutions of D, L α-T (Sigma), and β-C (Sigma) were used as standards. Results were expressed as pmol of α-T or β-C per milligram of wet weight.

The total content of MTs was quantified as described in Viarengo et al. (1997). This method included a precipitation of soluble proteins followed by the quantification of thiol groups (-SH). Samples (∼250 mg) were homogenized in a buffered solution of 20 mM Tris–HCl (pH 8.6) containing 0.5 M sucrose, 0.5 mM phenylmethylsulphonyl fluoride (peptidase inhibitor), and 0.01% *ß*-mercaptoethanol, and then were centrifuged at ×14,000 *g* for 40 min at 4°C. Proteins of each supernatant were precipitated with an ethanol/chloroform solution (107:8) followed by centrifugation at ×6,000 *g* for 10 min at 4°C; this step was repeated two times and then fractioned with acidic ethanol/chloroform (87:1). Pellets were suspended and solubilized in 5 mM Tris-HCl buffer with 1 mM EDTA (pH 7). MTs contents were quantified by the spectrophotometric assay 2,4-dinitrothiocyanobenzene (DTNB) and reduced glutathione (GSH) as standard. Results were expressed as nmol of thiol groups (-SH) per gram of wet weight.

Total uric acid concentration was measured according to Trinder (1969). Since the digestive gland of *P. canaliculata* accumulates uric acid in intracellular crystalloids (Vega et al., 2007), samples were homogenized in 0.5% lithium carbonate using an UltraTurrax^®^ homogenizer at 4°C. Homogenates were then centrifuged at ×3,000 *g* for 5 min at 4°C. Aliquots of 10 μL were treated with uricase, and the oxygen peroxide formed was used as substrate of the peroxidase enzyme to catalyze the reaction between 4-aminophenazone and chlorophenol. The colored quinoneimine product was quantified at 510 nm. The uric acid concentration was expressed as nmol of uric acid per milligram of wet weight.

#### 2.3.3 Enzymatic antioxidant defenses

To determine the enzymatic activities, digestive gland samples of approximately 120 mg were homogenized by sonication in a 1:4 (w/v) ratio in a buffer solution (20 mM Tris base, 1 mM EDTA, 1 mM dithiothreitol, 0.5 M sucrose, 0.15 M KCl, and 0.1 mM phenylmethylsulfonyl fluoride; pH 7.6). The homogenates were centrifuged at ×9000 *g* for 30 min (Bainy et al., 1996), and the supernatants were used for the determination of catalase (CAT) and glutathione-S-transferase (GST) enzymatic activities.

CAT activity was measured in 50 mM potassium phosphate buffer (pH 7) as described in Aebi (1984). The decomposition of H_2_O_2_ (50 mM) was measured at *λ* = 240 nm. A CAT unit was defined as the amount of enzyme that decompose 1 μmol of H_2_O_2_ in a min. Results were expressed as units (U) of CAT per milligram of protein.

GST activity was assayed using 50 mM of 1-chloro-2,4-dinitrobenzene (CDNB) as substrate in a reaction medium containing 100 mM GSH (Habig et al., 1974). The absorbance increase was measured at *λ* = 360 nm. A GST unit was defined as the amount of enzyme required to conjugate GSH with 1 μmol of CDNB per min. Results were expressed as milliunits (mU) of GST per milligram of protein.

Protein concentration was determined according to Lowry et al. (1951). Bovine serum albumin was used as standard.

#### 2.3.4 Lipid radicals (LR•) generation rate by electron paramagnetic resonance (EPR)

Digestive gland samples (∼25 mg) were homogenized manually in 150 µL of dimethyl sulfoxide containing 40 mM N-tert-butyl-α-phenylnitrone (PBN). Homogenates were incubated for 15 min at 37°C, and EPR spectra were obtained using a Bruker spectrometer ECS 106 (9.88 GHz with 50 kHz modulation frequency). EPR instrument settings for the spin trapping experiments were 19.56 mW of microwave power, 1120 G of modulation amplitude, 81.92 ms of time constant, and 2 × 10^4^ of receiver gain (Jurkiewicz and Buettner, 1994). Results were expressed as pmol of LR• per milligram of wet weight per minute (Kotake et al., 1996).

#### 2.3.5 Protein carboxylation

The increase of protein carboxylation by oxidative damage was determined by spectrophotometry as described in Levine et al. (1990). Samples (∼15 mg) were homogenized in potassium phosphate buffer (20 mM, pH 7.4) and then centrifuged at ×10,000 *g* for 30 min. The supernatants were incubated for 60 min in a 2 M HCl solution containing 10 mM 2, 4-dinitrophenylhydrazine (DNPH). The proteins were precipitated with 20% (w/v) trichloroacetic acid. Protein precipitates were washed three times with ethanol/ethyl acetate (1:1), and dissolved in a 20 mM potassium phosphate solution (pH 2.3) containing 6 M guanidine hydrochloride. Carbonyl groups (CG) content was determined by measuring the absorbance at *λ* = 360 nm after reaction to DNPH (Reznick and Packer, 1994). Protein content was measured according to Bradford (1976). Results were expressed as nmol of carbonyl groups per milligram of protein.

### 2.4 Statistical analysis

Our working hypothesis focuses on the search for cellular mechanisms activated after aquatic exposure to non-essential elements, implying that the phenomena studied occur spatiotemporally below individual level. Therefore, in our experimental sets (Sections 2.2 and 2.3), the digestive gland represents the observational unit while the individual snail constitutes the experimental unit. (Schank and Koehnle, 2009).

Elemental concentrations in the digestive gland from exposed and non-exposed snails at 96 h (Section 2.2) were compared with the Student’s *t*-test. These analyses were performed using GraphPad Prism 8.0.1^®^. The significance level was fixed at *p* < 0.05.

For each parameter evaluated in the digestive gland, multiple comparisons at different endpoints (0, 24, 48, 72, and 96 h) into each category (control, Hg, As, or U) were made using Generalized Linear Models (GLMs). When significant (*p* < 0.05), the Least Significant Difference (LSD) of Fisher was applied. Data analyses were performed using STATGRAPHICS Centurion XVI (version 16.0.07).

The descriptive analysis and *p*-values are reported in Supplementary Data S3.

## 3 Results

**FIGURE 1:**
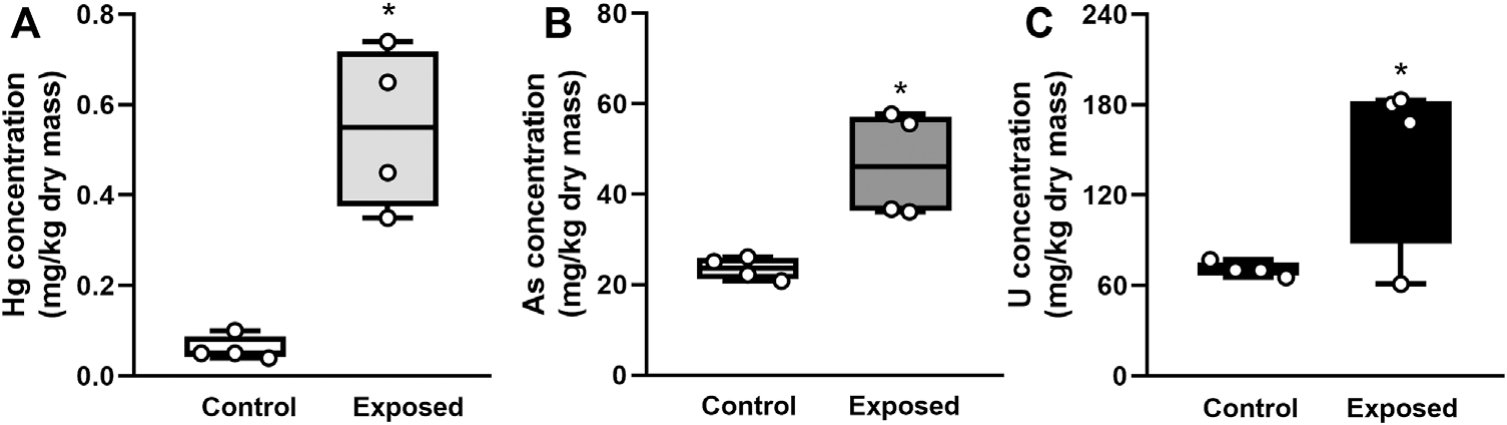
Elemental concentration in the digestive gland in snails exposed for 96 h to Hg **(A)**, As **(B)** or U **(C)**. Mean ± SEM was computed for each group. Asterisks indicate statistically significant differences between control and exposed animals (Student’s *t*-test).

Elemental concentrations of Hg, As, and U in digestive gland were significantly higher in exposed snails than in non-exposed snails (Student’s *t*-test, *p* < 0.05; Figure1). Bioconcentration factors of Hg, As, and U were 25, 23, and 53, respectively.

**FIGURE 2:**
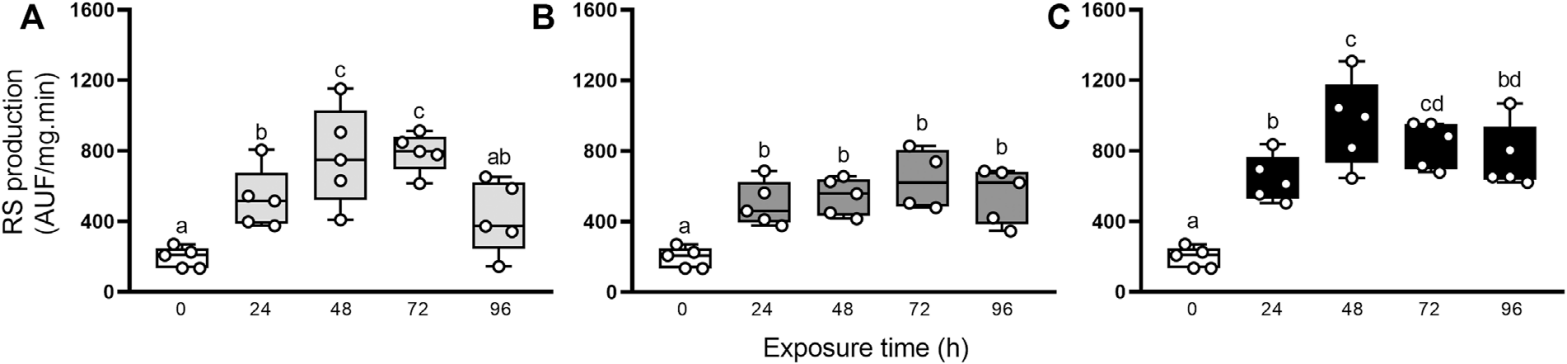
RS production in the digestive gland from exposed snails to 5.5 μg/L of Hg **(A)**, 500 μg/L of As **(B)**, or 700 μg/L of U **(C)**. Mean ± SEM was computed for each group. Different letters indicate statistically significant differences at different times after exposure to the same element (GLM; Least Significant Difference of Fisher).

To characterize the oxidative state, we first evaluated the production of RS (Figure 2). This parameter significantly increased in the first 24 h after elemental exposure (Figure 2A–C, respectively), compared to group control (0 h), and it remained significantly high until the end of the experimental period (72 h for Hg and 96 h for As and U).

**FIGURE 3:**
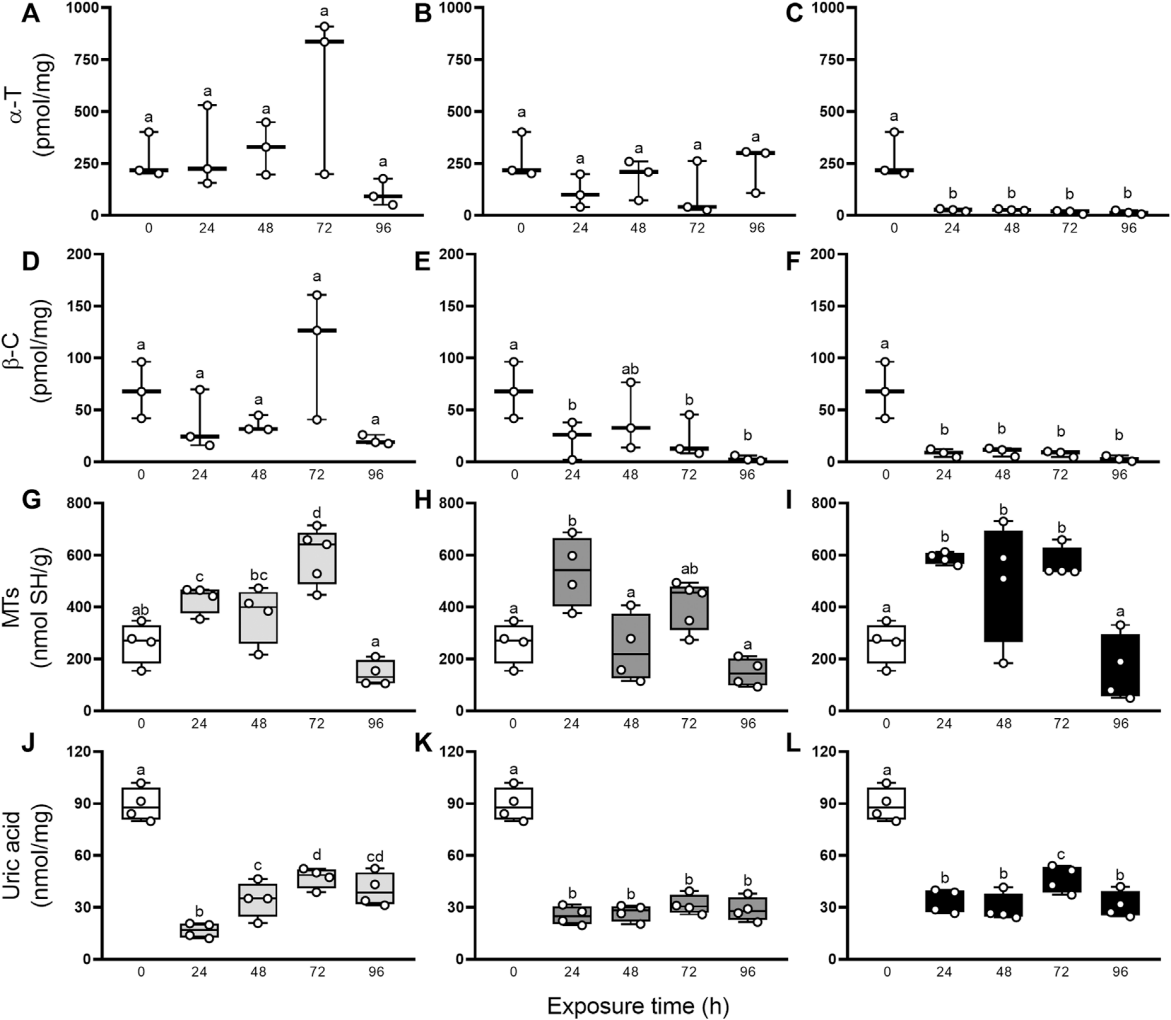
α-T **(A–C)**, β-C **(D–F)**, MTs **(G–I)**, and uric acid **(J–L)** concentrations in digestive gland from exposed snails to 5.5 μg/L of Hg (light gray), 500 μg/L of As (dark gray), or 700 μg/L of U (black). Mean ± SEM was computed for each group. Different letters indicate statistically significant differences at different times after exposure to the same element (GLM; Least Significant Difference of Fisher).

**FIGURE 4:**
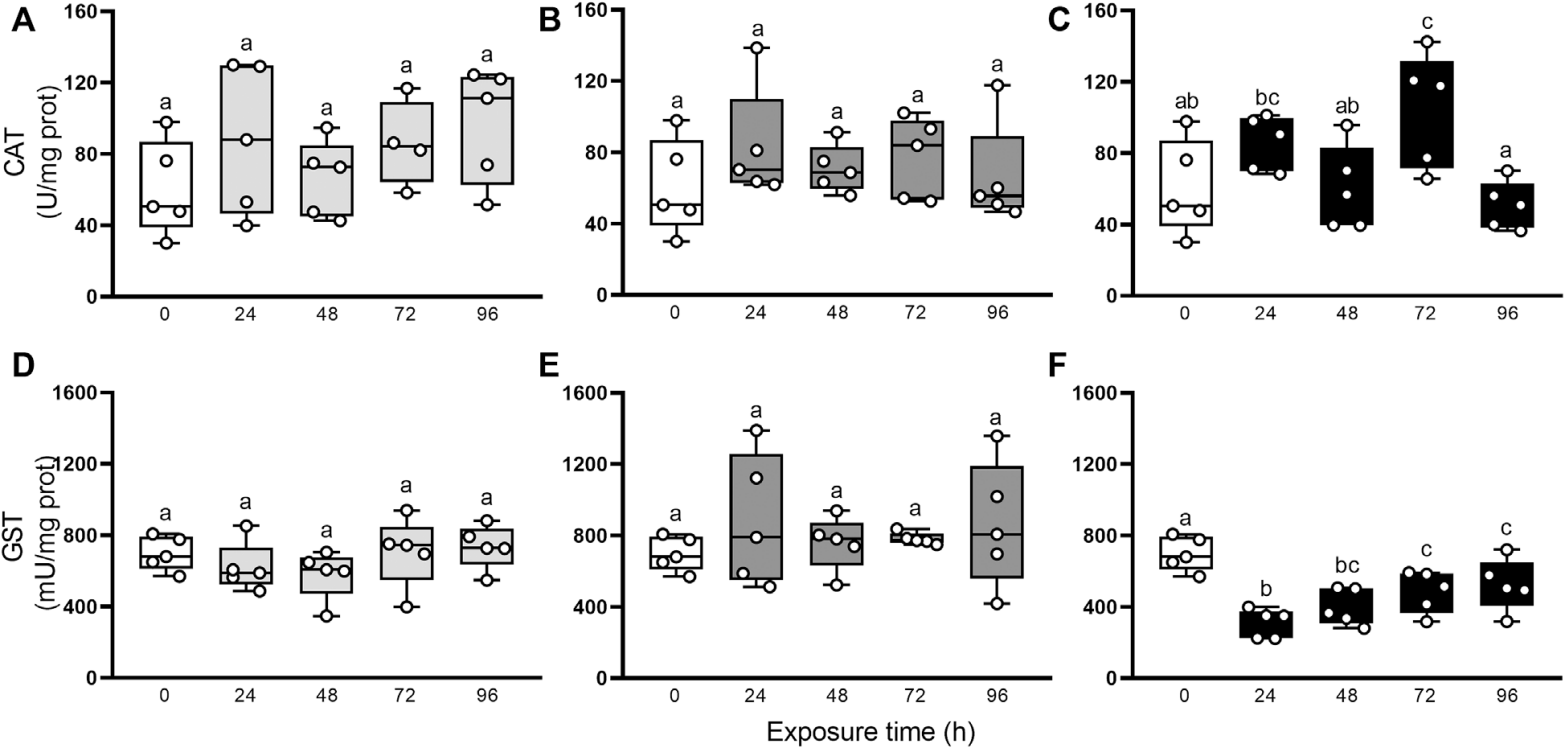
CAT **(A–C)** and GST **(D–F)** activities in digestive gland from exposed snail to 5.5 μg/L of Hg (light gray), 500 μg/L of As (dark gray), or 700 μg/L of U (black). Mean ± SEM was computed for each group. Different letters indicate statistically significant differences at different times after exposure to the same element (GLM; Least Significant Difference of Fisher).

Besides, the characterization of the oxidative state was completed with the evaluation of non-enzymatic and enzymatic antioxidants defenses. Figure 3 resumes the involvement of non-enzymatic antioxidant defenses after elemental exposure. In snails exposed to Hg, the α-T and β-C levels did not change significantly (GLM, *p* > 0.05; Figure 3A, D). In As-exposed animals, α-T level remained unchanged (GLM, *p* > 0.05; Figure 3B) but β-C concentration fell significantly in the first 24 h, compared to control (0 h), then recovered to levels close to basal at 48 h to end up falling until the end of the exposure again (Figure 3E). In U-exposed snails, α-T and β-C concentrations were consumed early (24 h) and remained significantly low until 96 h (Figures 3C, F). Digestive gland showed a significant increase in MTs after elemental exposure (Figures 3G–I): 24 and 72 h in Hg-exposed snails, 24 h in As-exposed snails, and 24, 48 and 72 h in U-exposed snails. Total uric acid concentrations (Figures 3J–L) decreased significantly after 24 h exposure to Hg, As, and U, and remained low until the end of the exposure time (96 h). Figure 4 shows the enzymatic antioxidant defenses after elemental exposure. Both enzymatic activities, CAT and GST, remained unchanged in Hg-exposed (GLM, *p* > 0.05; Figures 4A, D) and As-exposed (GLM, *p* > 0.05; Figure 4B, E) snails. In U-exposed animals, CAT activity increased significantly at 72 h, compared to other endpoints, (Figure 4C), while GST activity dropped abruptly at 24 h (Figure 4E) and it began to increase continuously until the end of the exposure (72 and 96 h); although this increase fails to restore its pre-exposure values.

**FIGURE 5:**
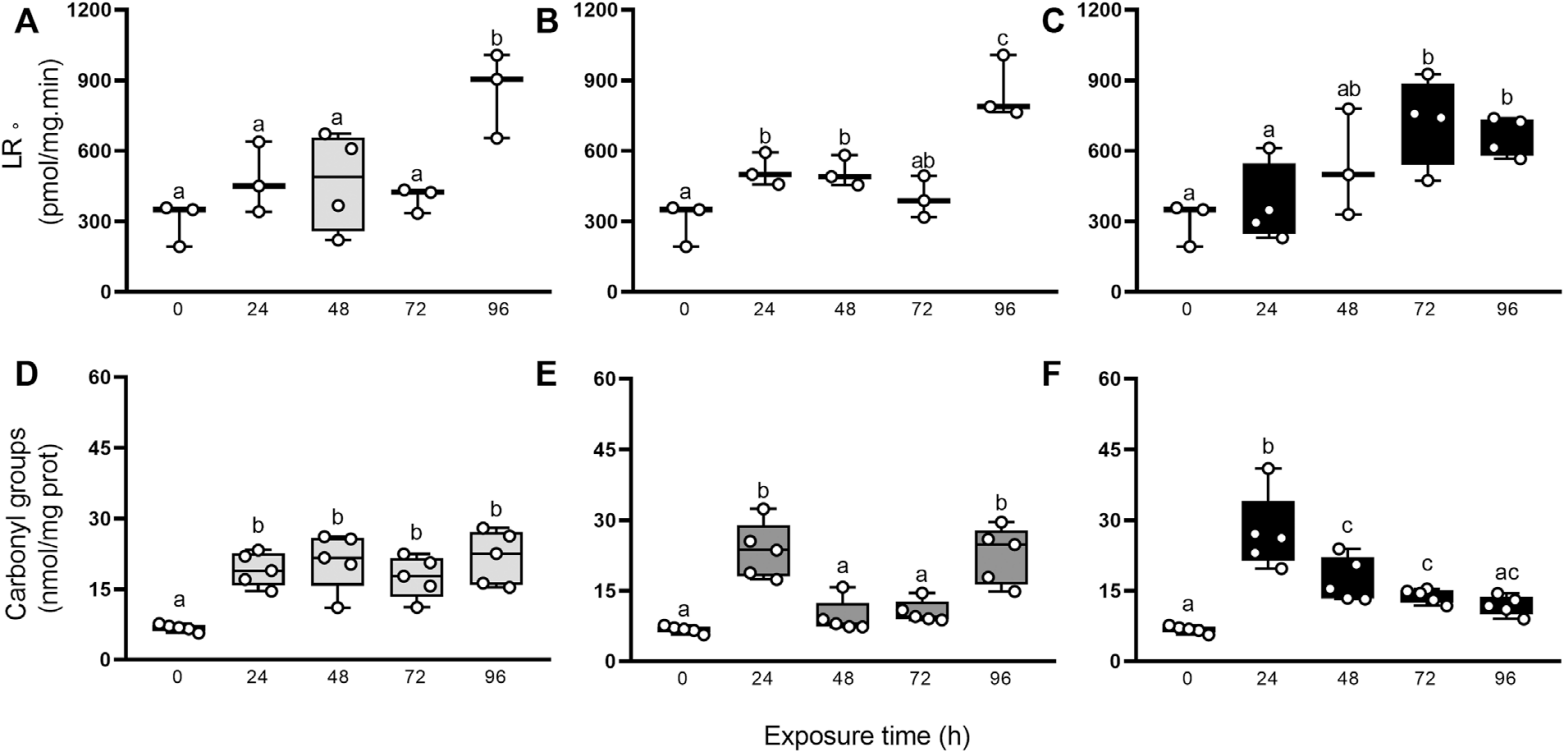
LR• generation rate **(A–C)**, and carbonyl groups concentration **(D–F)** in digestive gland from exposed snails to 5.5 μg/L of Hg (light gray), 500 μg/L of As (dark gray), or 700 μg/L of U (black). Mean ± SEM was computed for each group. Different letters indicate statistically significant differences at different times after exposure to the same element (GLM; Least Significant Difference of Fisher.

Additionally, we evaluated if the antioxidant protection mechanisms were sufficient to counteract RS production after elemental exposure through the occurrence of damage to biomolecules such as lipids and proteins (Figure 5). The generation rate of LR• and carbonyl groups were higher in exposed animals compared to control group (0 h), but statistical significance was reached only at some endpoints. The GLMs analysis showed significantly higher values of LR• for Hg, (96 h > 0, 24, 48, 72 h), As (96 h > 24 and 48 h > 0 h), and U (24 h > 48 and 72 h > 0 h), and of carbonyl groups for Hg (24–96 h > 0 h), As (24 and 96 h > 24 and 48 h > 0 h), and U (24 h > 48 and 72 h > 0 h).

## 4 Discussion

The central objective of the current work was to assess changes in the oxidative status and lipids and proteins oxidation in the digestive gland of the gastropod *Pomacea canaliculata* after an aquatic exposition for 96 h to ecologically relevant concentrations of Hg, As, and U.

The animals did not show mortality during exposure, despite the large concentrations of these elements, which are toxic to aquatic ecosystems and human health. Our preceding studies (Vega et al., 2012; Campoy-Diaz et al., 2018; Juarez et al., 2022) showed that the digestive gland accumulates metals of class A, B, and intermediate from water. Moreover, digestive epithelial cells may accumulate elements directly by endocytosis after food digestion in the stomach (Juarez et al., 2022). Herein, the digestive gland bioconcentrates the three non-essential elements, generating early reactive species thus causing protein and lipid oxidation. Time changes in the lipid peroxidation and protein damage differed among the three elements used (Figure 5). It is likely that this cellular response is associated with the affinities of Hg, As, and U for different functional biological groups or with different degrees of effectiveness of antioxidant defenses. Inorganic Hg and As have a high affinity for reduced sulfur groups (Sharma et al., 1993; Quig, 1998; Ravichandran, 2004; Shen et al., 2005; Maltez et al., 2009), depleting intracellular reserve of thiols (Valko et al., 2005). Furthermore, As and U (in the form of uranyl ion) are prooxidant elements that may respectively increase the superoxide anion and hydrogen peroxide levels (Shi et al., 2004), hydroxyl radicals, and hydrogen peroxide (Garmash et al., 2014), thus producing oxidative stress and macromolecules damage (Barescut et al., 2005; Bergmann et al., 2018). Additionally, uranyl may bind to nucleotides through their phosphoric groups, competing with calcium and magnesium ions, which cause a reduction of bioavailable ATP for different metabolic processes (De Stefano et al., 2005). Besides, U may deplete reduced glutathione, as was reported in isolated hepatocytes from laboratory rat (*Rattus norvegicus*, Pourahmad et al., 2011) and zebrafish (*Danio rerio*, Barillet et al., 2011).

Given that the three elements produce an oxidative imbalance in the digestive gland of *P. canaliculata*, snails may effectively delay or attenuate lipid peroxidation and protein damage activating its cellular antioxidant machinery, as occurs against other environmental stressors such as low temperature, desiccation, or xenobiotics (Giraud-Billoud et al., 2011; Giraud-Billoud et al., 2013; Giraud-Billoud et al., 2018; Giraud-Billoud et al., 2022). In the next paragraphs, we discuss the importance and effectiveness of each antioxidant defense during oxidative imbalance.

α-T and β-C are lipophilic dietary molecules currently known in mollusks, with behavioral, physiological, and protective functions (Tsushima et al., 1997; Dreon et al., 2004; Rivera-Ingraham et al., 2013; Abdullah and Seulalae, 2020). These molecules are often associated with cell membranes and prevent lipid peroxidation by neutralizing specific reactive species (Neely et al., 1988; Sies and Stahl, 1995) or even interrupting the sequential oxidation reactions of polyunsaturated fatty acids (Scarpa et al., 1984; Sies and Stahl, 1995). Herein, the consumption of α-T and β-C was variable among snails exposed to three elements. U-exposed snails showed an early and high consumption of both molecules, counteracting partially the oxidative burst and delaying the lipid peroxidation. In As-exposed snails, the consumption of β-C was around 67%, but that of α-T was not significant during the entire experimental period, which could be indicating a compartmentalization of the oxidative process. Interestingly, Hg-exposed snails maintained the levels of α-T and β-C without changes in lipid peroxidation during the first 72 h. These findings may be associated with the fact that the digestive gland of *P. canaliculata* is the main organ involved in the acquisition, accumulation, and metabolization of different dietary carotenoids (Tsushima et al., 1997), and that the individuals were fed throughout the experimental period (this work).

MTs are cytosolic non-enzymatic cysteine-rich proteins virtually present in all animals (Blindauer and Leszczyszyn, 2010) that regulate the availability of essential metals (Zn and Cu), and detoxify non-essential elements (Amiard et al., 2006). In this work, high levels of MTs were reached after elemental exposure, but with different kinetics. It is likely that these cellular changes are associated with a differential expression, as was reported in the apple snails *Pomacea bridgesii* and *Marisa cornuarietis* exposed to intermediate and B-class elements (Maltez et al., 2009). Also, the MTs variations may be a consequence of their lysosomal compartmentalization (Hamer, 1986) for its subsequent degradation and excretion (Marigomez et al., 2002). This evaluation should be considered a preliminary appraisal and a complete one should evaluate the transcription of two MTs codifying genes found in the genome in *P. canaliculata* (Liu et al., 2018; Sun et al., 2019).

*P. canaliculata* accumulates urate crystalloids in the perivascular tissue of the digestive gland (Vega et al., 2007; Giraud-Billoud et al., 2008). Previous evidence has shown that uric acid-producing cells exhibit an active turnover between asynchronous formation and lysis of crystalloids (Giraud-Billoud et al., 2008). Furthermore, uric acid is an antioxidant in this species, used after long periods of dormancy induced by estivation or hibernation to tolerate the oxidative damage induced by arousal (Giraud-Billoud et al., 2013; Giraud-Billoud et al., 2018). In this work, total uric acid decreased approximately 3-fold 24 h after exposure to Hg, As, and U, and it did not recover the initial levels, which may indicate an increase in crystalloid lysis and its use as a non-enzymatic antioxidant to face the oxidative burst. It is possible that uric acid and carotenoids act as the first line of defense against the increase in RS induced by exposure to metals, to prevent lipid peroxidation of cell membranes.

CAT is an antioxidant enzyme that catalyzes the reduction of intracellular H_2_O_2_ to water and oxygen (Orbea et al., 2000), thus counteracting the activity of H_2_O_2_-generating oxidases. The role of CAT, as an enzymatic antioxidant protective mechanism in *P. canaliculata*, has recently been characterized in models of acute stress where reactivation after hypometabolism induced by estivation or hibernation is related to increases in its expression and activity (Sun et al., 2019; Giraud Billoud et al., 2022; Rodriguez et al., 2023). However, the enzymatic activity of CAT remains approximately constant (with some small variations in U-exposed snails), even though the non-enzymatic antioxidant defenses partially counteract RS and protein damage. It is still possible that the protein synthesis or CAT activity may be affected by the presence of metals in the digestive gland cells. The GST family includes cytosolic enzymes that conjugate electrophile groups with reduced glutathione for detoxification (Bhagat et al., 2016). These enzymes do not seem to have a functional role in snails exposed to the three elements studied. Moreover, the GST activity falls in U-exposed snail, which may be associated with early protein damage. Together, these findings could be indicating that CAT and GST are not necessary at the early stages of the oxidative process induced by metals.

## 5 Conclusion

The freshwater gastropod *P. canaliculata* bioconcentrated Hg, As, and U at high levels in the digestive gland when exposed for 96 h at ecologically relevant concentrations. These elements generated early RS, lipid peroxidation, and the protein damage and redox disbalance were partially compensated by non-enzymatic antioxidant defenses. The consumptions of α-T, β-C, and uric acid were variable among snails exposed to three elements. MTs increased at similar levels after exposure but with different kinetics. CAT and GST were not necessary for the early stages of the oxidative process by metals. The bioconcentration factors and biochemical parameters studied in this work confirm that this species may be used as a bioindicator of metal pollution in freshwater bodies. Two ecotoxicological considerations related to human and animal health should be noted: 1) the direct consumption of apple snails by humans or their use as feed for pigs, and 2) the possible biomagnification of metals in ecosystems, where birds almost exclusively consume these snails.

## Data Availability

The raw data supporting the conclusions of this article is available as Supplemental Materials (Tables S1 and S2).
